# The Genome Sequence of *Weissella cibaria* DmW_103, Isolated from Wild *Drosophila*

**DOI:** 10.1128/genomeA.00512-17

**Published:** 2017-06-15

**Authors:** Nathan J. Ricks, Courtney Carroll, Amber Walters, Peter D. Newell, John M. Chaston

**Affiliations:** a Department of Plant and Wildlife Sciences, Brigham Young University, Provo, Utah, USA; b Department of Biological Sciences, SUNY Oswego, Oswego, New York, USA

## Abstract

Lactic acid bacteria are commonly associated with *Drosophila* spp. Here, we report on the isolation of a strain of *Weissella cibaria* and the sequencing, assembly, and annotation of its genome. A total of 35 contigs were generated, with 2,349 coding sequences found.

## GENOME ANNOUNCEMENT

*Weissella cibaria* is a lactic acid–producing bacterium that has potential uses in many industries, ranging from food production to medicine. For example, *W. cibaria* produces bacteriocins that have been studied for potential use as meat and dairy preservatives (1). Also, *W. cibaria* isolates have been screened for both general probiotic properties (adhesion and bile salt resistance) (2) and for conferring protection against a specific skin disease, atopic dermatitis (3). Beyond these properties, lactic acid bacteria are often associated with *Drosophila* spp. and influence their life history traits (4, 5). Since most genome sequences for lactic acid bacteria isolated from *Drosophila* spp. are restricted to a single genus (*Lactobacillus*), we sought to expand this taxonomic range by sequencing the genome of *W. cibaria* isolated from wild *Drosophila*.

DNA from *W. cibaria* was extracted and assembled using the following methods. Wild *Drosophila* samples were collected from compost in Ithaca, New York, USA (42.427447°N, 76.464339°W), homogenized, and diluted onto modified MRS medium (6). A single colony was isolated, and its identity was verified by Sanger sequencing of the 16S rRNA gene. To prepare the DNA for whole-genome shotgun sequencing with a 1,200-bp insert size, DNA was extracted using the Qiagen DNeasy Blood & Tissue Kit and fragmented by NEB fragmentase, and the adapters were ligated using components of the NEBNext Ultra II Ligation Module Kit according to manufacturer’s instructions. The library was sequenced using paired-end 250-bp sequencing chemistry on an Illumina HiSeq 2500; 11,001,772 reads passed quality filtering, and the sequence assembly proceeded using Velvet version 1.2.10, as in our previous work (7, 8). The reads were randomly divided into 13 bins, each representing 200× genome coverage, and for each bin a separate genome sequence was assembled by selecting the *k*-mer length between 191 and 211 that maximized the *N*_50_ (range of 150,723 to 160,231 bp, mean 157,625 bp). A consensus *W. cibaria* DmW_103 genome was assembled using the representative contig file produced in each bin’s separate assembly. A final assembly of 2,458,382 nucleotides in 35 contigs had an *N*_50_ of 160,221 and a max contig length of 434,968. ANIm analysis against *Weissella* spp. in the JSpeciesWS database in April 2017 confirmed the genome was from a *W. cibaria* isolate (>95% identity) (9). The data were submitted to GenBank and annotated by the NCBI Prokaryotic Genome Annotation Pipeline, yielding 2,349 genes.

Preliminary investigation using annotations produced in RAST (10–12) identified metabolic pathways unique to *W. cibaria*. Related to the other two *Leuconostocaceae* spp. with publically available genomes in RAST, only *W. cibaria* had annotated genes with functions in glycerate, glycogen, or sialic acid metabolism, or in fructose utilization. It would be interesting to test in a future work if any of these differentially present pathways influence *D. melanogaster* metabolism.

### Accession number(s).

The whole-genome shotgun data have been deposited in GenBank under the accession number NDXJ00000000. The version described in this paper is the first version, NDXJ01000000.
